# Isolated Abdominal Aortitis Following a Urinary Tract Infection

**DOI:** 10.7759/cureus.18902

**Published:** 2021-10-19

**Authors:** Ala Mustafa, Pablo Weilg, Larry Young, Christopher Anzalone, Denisa Hagau

**Affiliations:** 1 Internal Medicine, MercyOne North Iowa Medical Center, Mason City, USA; 2 Rheumatology, Boston Medical Center, Boston, USA; 3 Rheumatology, University of Miami, Coral Gables, USA; 4 Cardiology, MercyOne North Iowa Medical Center, Mason City, USA

**Keywords:** isolated, aortitis, uti, idiopathic aortitis, infectious aortitis

## Abstract

A 49-year-old female with a history of sporadic episodes of scleritis was initially seen by her primary care physician (PCP) due to a two-day history of cramping abdominal pain, new elevated high blood pressure, increased urinary frequency, and urgency. The patient was diagnosed with an acute cystitis supported by a positive urine culture for a pan sensitive *Escherichia coli*; however, after two courses of antibiotics as an outpatient, her blood pressure (BP) remained markedly elevated, and her abdominal pain got worse which prompted a computed tomography (CT) abdomen and pelvis with contrast revealing inflammatory changes consistent with aortitis. The diagnosis was supported by a magnetic resonance angiography (MRA) which showed wall thickening and enhancement extending for approximately 4.8 cm involving the abdominal aortic wall just prior to the bifurcation. An extensive work up including CTA, US doppler of four-limbs, and fluorodeoxyglucose (FDG)-positron emission tomography (PET) confirmed the isolated abdominal aortitis. After infectious etiologies were ruled out, the patient was started on prednisone 60 mg daily which resulted in marked improvement of her symptoms. After a four-month taper of steroids, the patient had complete resolution of her symptoms, with no signs of recurrence.

## Introduction

Aortitis is a non-specific term that describes the inflammation of any of the aortic wall layers irrespective of the underlying etiology [1,2]. Several conditions have been associated with the development of aortitis including infectious causes, as well as connective tissue diseases [1-3]. In addition, idiopathic (isolated) aortitis has been described in up to 8.4% of all cases of aortitis [1,4,5]. The pathophysiology of these idiopathic vasculitides is not entirely understood; however, various mechanisms have been proposed to be involved in the relationship between infections and systemic vasculitis [1,6-8]. Furthermore, risk factors such as tobacco smoking, young or advanced age at presentation, history of connective tissue disease, diabetes, heart pathology, or family history of aortic aneurysm have been associated with an increased risk for aortitis [1,6].

## Case presentation

A 49-year-old Caucasian woman with a past medical history significant for sporadic episodes of scleritis, with her last episode six years ago, presented to her primary care physician’s (PCP) office due to a two-day history of abdominal pain associated with increased urinary frequency and urgency. The abdominal pain was described as cramping lower abdominal, rating it at a six of 10 with no radiation, aggravating, or alleviating factors. She denied any fever, chills, nausea, vomiting, dysuria, hematuria, or vaginal discharge. Other pertinent negatives included headache, jaw claudication, dizziness, vertigo, night sweats, fatigue, chest pain, palpitations, shortness of breath, eye pain, vision changes, weakness, numbness, oral or genital ulcers, or joint pain/swelling. The PCP physical examination revealed lower abdominal tenderness as well as new hypertension with a right arm blood pressure (BP) of 177/100 mmHg. A urinalysis (UA) showed clear yellow urine with white blood cell (WBC) 25 cells/hpf, red blood cell (RBC) 3-5 cells/hpf, bacteria +3, and nitrites positive. The patient was diagnosed with acute simple cystitis and was started on trimethoprim/sulfamethoxazole DS 800-160 mg twice daily for a total of 10 days. The elevated BP was considered an isolated event and no antihypertensive medication was started at that time.

**Figure 1:**
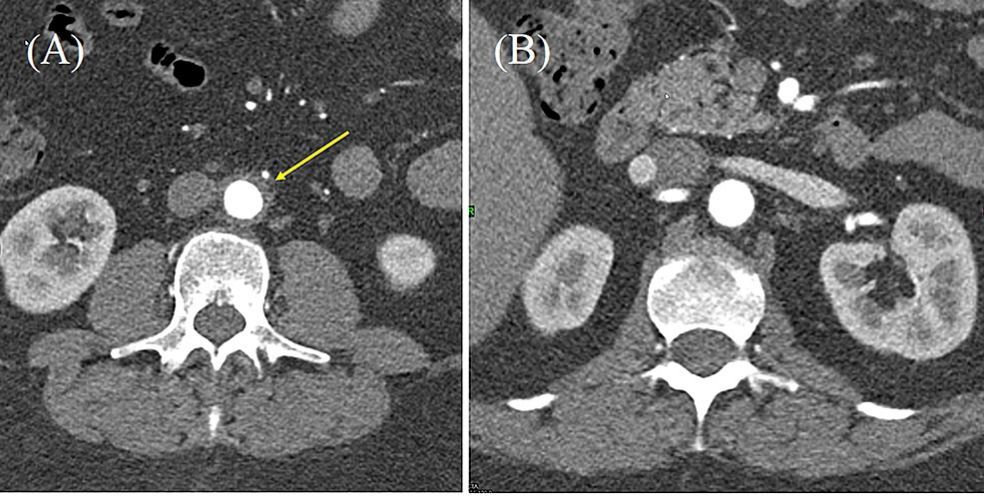
CT abdomen and pelvis with contrast (A) Mid-abdominal aorta with signs of wall thickening and adjacent inflammation (arrow); (B) level of upper abdominal aorta without signs of wall thickening and inflammation.

At her follow-up appointment, two weeks later, she reported no improvement in her urinary frequency and urgency. However, her abdominal pain was not as bothersome, and an improved BP of 138/82 mmHg was noted. The urine culture from the previous visit showed pan-sensitive *Escherichia coli*, and a repeat UA at this visit was negative for pyuria, hematuria, or bacteriuria. The patient’s PCP felt it necessary to continue antibiotic treatment due to persistent symptoms despite the normal UA, and she was started on cephalexin 500 mg three times daily for seven days. At the four-week follow-up, she complained of worsening frequency and intensity of her cramping abdominal pain. Her blood pressure was noted to be more elevated on this occasion. At this time, a comprehensive metabolic panel (CMP), complete blood count (CBC), and computed tomography (CT) of the abdomen and pelvis with contrast were ordered by her PCP. While her CBC and CMP were unrevealing (Table 1), her CT showed marked mucosal enhancement of the urinary bladder with prominent peri-vesical inflammation suggesting cystitis and mild-to-moderate inflammatory changes surrounding the distal abdominal aorta suggesting aortitis (Figures 1A, 1B). Immediately after the results were available, the patient was contacted and informed to go to the emergency department (ED).

**Table 1:** Complete blood cell count and comprehensive metabolic panel WBC: white blood count; RBC: red blood count; BUN: blood urea nitrogen; GFR: glomerular filtration rate; AST: aspartate aminotransferase; SGOT: serum glutamic oxaloacetic transaminase; ALT: alanine transaminase; SGPT: serum glutamic pyruvic transaminase

CBC with differential	Unit
WBC count	8.46 × 10^3^/mL
RBC Count	4.54 × 10^6^/mL
Hemoglobin	12.6 g/dL
Hematocrit	39.3%
Platelet	227 ×10^9^/L
Comprehensive metabolic panel	
Sodium	137 mmol/L
Potassium	3.6 mmol/L
Chloride	104 mmol/L
Carbon dioxide	27 mmol/L
Glucose	108 mg/dL
BUN	12 mg/dL
Creatinine	1 mg/dL
Estimated GFR	59.2 mg/min/BSA
Calcium	8.7 mg/dL
Bilirubin	0.3 mg/dL
AST/SGOT	15 U/L
ALT/SGPT	14 U/L
Creatinine	1 mg/dL
Estimated GFR	59.2 mg/min/BSA
Calcium	8.7 mg/dL
Bilirubin	0.3 mg/dL
AST/SGOT	15 U/L
ALT/SGPT	14 U/L
Creatinine	1 mg/dL
Estimated GFR	59.2 mg/min/BSA
Calcium	8.7 mg/dL
Bilirubin	0.3 mg/dL

**Figure 2:**
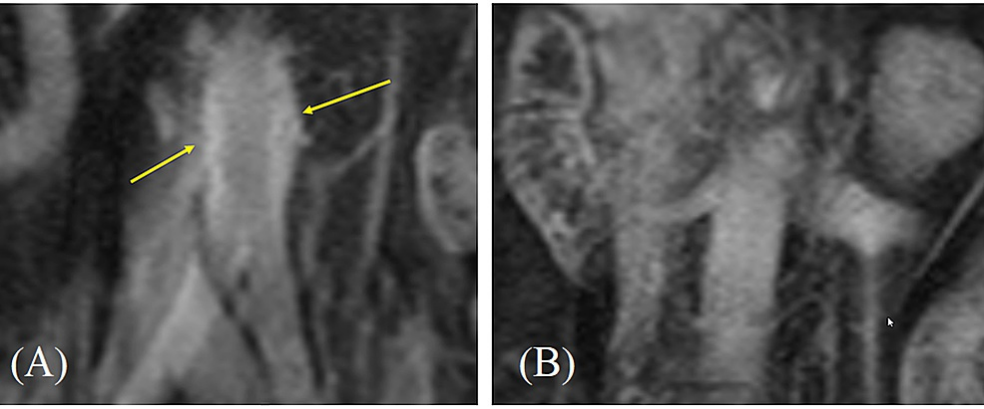
MRA chest, abdomen, and pelvis with and without contrast Coronal view of abdominal aorta (A) at bifurcation post-contrast with (arrows) and (B) at the level of the right renal artery without wall thickening, inflammation, and contrast enhancement. MRA: magnetic resonance angiography

Once in the ED, further laboratory workup was performed which was significant for an erythrocyte sedimentation rate (ESR) of 38 mm/h and UA showing recurrent pyuria and bacteriuria with WBC 26 cells/hpf, RBC 0-2 cells/hpf, bacteria 1+, and positive leukocyte esterase. The patient continued to be persistently hypertensive at 182/98 mmHg, with no significant difference between readings on her arms. A magnetic resonance angiography (MRA) of the chest abdomen and pelvis with and without contrast showed wall thickening and enhancement extending for approximately 4.8 cm involving the abdominal aortic wall just prior to the bifurcation, suggestive of a large vessel vasculitis, with no other vessel involvement (Figures 2A, 2B). Additionally, a CT angiography of the head and neck with and without contrast as well as a duplex carotid ultrasound were also negative for vascular involvement.

The patient was admitted to the hospital where she was started on labetalol 10 mg IV every four hours as needed for BP control and ciprofloxacin 400 mg IV every 12 hours given her previous antibiotic courses. She was evaluated by the infectious disease (ID) departments which after further workup deemed the etiology of her aortitis unlikely to be infectious (Table 2). She was started on prednisone 60 mg orally daily for her isolated aortitis by the rheumatology department. Cardiology started the patient on lisinopril 10 mg orally daily with adequate control of her BP. Following two days of treatment, the patient had significant improvement in her symptoms and her BP remained controlled with lisinopril 10 mg orally daily.

**Table 2:** Rheumatologic workup Ag: antigen; CRP: C-reactive protein; Ab: antibody; dsDNA: antidouble stranded DNA; Sm: smith; c-ANCA: antineutrophil cytoplasmic antibodies; p-ANCA: perinuclear antineutrophil cytoplasmic antibodies; HCV: hepatitis C virus; HBc: hepatitis B core; RNP: ribonucleoprotein; HBs: hepatitis B surface antibody

Rheumatologic workup	
Histoplasma antigen, urine	Negative
Histoplasma mycelial	Negative
Histoplasma yeast	Negative
Histoplasma immunodiffusion	Negative
Histoplasma Ag value	0 U
Sedimentation rate, B	5 mm/h
CRP	<3.0 mg/L
Rheumatoid factor, S	<15 IU/mL
Cyclic citrullinated peptide, Ab, S	<15.6 U
Antinuclear Ab, Hep-2 substrate, S	<1:80 (Negative)
dsDNA Ab with reflex, IgG, S	<12.3 IU/mL
SS-A/Ro Ab, IgG, S	<0.2 U
SS-B/Ro Ab, IgG, S	<0.2 U
Sm AB, IgG, S	<0.2 U
RNP Ab, IgG, S	<0.2 U
Scl 70 Ab, IgG, S	<0.2 U
Jo 1 Ab, IgG, S	<0.2 U
c-ANCA	Negative
p-ANCA	Negative
Myeloperoxidase Ab, S	<0.2 U
Proteinase 3 Ab (PR3), S	<0.2 U
Complement C3, S	105 mg/dL
Complement C4, S	22 mg/dL
Immunoglobulin subclass IgG4, S	8.6 mg/dL
6-Methylmercaptopurine	4.44 nmol/mL/h
6-Methylmercaptopurine riboside	6.17 nmol/mL/h
6-Methylthioguanine riboside	4.79 nmol/mL/h
Complement, total	64 U/mL
HBs antigen, S	Negative
HBs antibody, S	Negative
HBs antibody, quantitative, S	<5.0 mIU/mL
HBc total Ab, S	Negative
HCV Ab, S	Negative
Quantiferon-TB gold plus	Negative
Syphilis total Ab w/reflex	Nonreactive

The patient had two sets of blood cultures reported as negative after five days and her urine culture returned positive again for pan-sensitive *E. coli*. Thus, ID recommended completing a total of five days of ciprofloxacin 500 mg orally twice a day for a urinary tract re-infection. She was discharged on prednisone 60 mg daily with a decreasing taper of 20 mg every two weeks, a close follow-up with her PCP within five days, and with rheumatology within two to three weeks. Two weeks after her discharge, four limb arterial doppler testing showed a normal study along with similar BP readings. Furthermore, a fluorodeoxyglucose (FDG)-positron emission tomography (PET) showed improvement of her aortitis while on 40 mg of prednisone daily. On that occasion, she also denied any residual abdominal or urinary symptoms, and a decision was made to continue her prednisone taper as follows: 15 mg for two weeks, 10 mg for two weeks, 7.5 mg for two weeks, 5 mg for four weeks, and 2.5 mg for four weeks. Three months later, at her last follow-up appointment, the patient had completed her prednisone course with no evidence of relapse of her aortitis. No disease-modifying agent with steroid-sparing medication were started. She will continue to follow up with the rheumatology services in three months.

## Discussion

Idiopathic aortitis (IA) is an underrecognized form of vasculitis characterized by the presence of isolated inflammation of the aorta without any identifiable systemic disease or infectious source. The initial assessment of a patient with aortitis requires prompt identification of an infectious etiology due to its rapid progression, complications, and high mortality [2,9]. Conversely, non-infectious aortitis cases typically present with a protracted course which may result in the development of aortic stenoses and aneurysm [2,3]. Furthermore, cases of aortitis often remain undetected until they are identified at later stages [8]. In patients with a newly diagnosed aortitis, non-specific clinical features have been reported such as fever, headaches, chest or abdominal pain, vascular insufficiency, myalgias, arthralgias, among others [2,8].

Our patient presented with cramping abdominal pain associated with urinary frequency and urgency. She was initially diagnosed with an acute simple cystitis based on her urinary symptoms and a positive urinalysis for pyuria and bacteriuria. The diagnosis of acute simple cystitis was later supported with a positive urine culture with more than 100,000 CFU for a pan-sensitive *E. coli*. However, due to her persistent symptoms, she received two courses of antibiotics. After four weeks, the progression of her abdominal pain radiating to her back associated with newly diagnosed hypertension prompted a CT abdomen and pelvis with contrast, which revealed marked mucosal enhancement of the urinary bladder compatible with cystitis as well as moderate inflammatory changes surrounding the distal abdominal aorta suggesting aortitis (Figure 1). Additionally, an MRA of the abdomen with and without contrast also reported wall thickening and enhancement extending for approximately 4.8 cm further supporting the evidence of abdominal aortitis.

Infectious aortitis most commonly occurs due to hematogenous seeding of an existing intimal injury, but cases of septic emboli, direct spread from an infective focus, and bacterial inoculation have been reported [2]. The most common causes of bacterial aortitis are Staphylococcus spp. and Salmonella spp. [2,10]. Our patient did not have any strong risk factors for either of these pathogens, and two negative blood cultures argue against a bacterial source, though it is important to mention that the patient had two courses of oral antibiotics before the blood culture was collected which could have made the bacterial isolation in the blood more challenging. Other etiologies were less likely to be the culprit due to the negative serology for syphilis, Histoplasma, HIV, and hepatitis B and C. In addition, the patient had a negative QuantiFERON Gold and a negative history of tuberculosis exposure.

Nonetheless, we cannot ignore the presence of an *E. coli* urinary tract infection concomitantly presenting with abdominal aortitis. *E. coli* is not typically a common cause of aortitis; however, one case report of primary aortitis has been reported as sequelae of an incompletely treated urinary tract infection [11]. This case had a similar history interval of three weeks, but that patient predominantly presented with fever, chills, and abdominal pain; he required an axillofemoral bypass with resection of the infected aorta along with a six-week course of intravenous antibiotics highlighting the severity of most infective aortitis cases. On the contrary, in our case, there was clear evidence of a negative urinalysis and urine culture after the first course of antibiotics, followed by a re-infection with the same pathogen four weeks later leading to a third course of oral antibiotics with ciprofloxacin for five days. Additionally, the patient had two sets of blood cultures reported as negative after five days. We believe that the resolution of the symptoms after the administration of steroids coupled with the lack of further complications in our follow-up visits suggests a non-infectious etiology since a case of bacterial aortitis would not be expected to resolve after three short courses of oral antibiotics. Nonetheless, several mechanisms have been proposed to be involved in the relationship between infections and the development of systemic vasculitis including molecular mimicry, superantigens, cell activation by Toll-like receptors (TLR), among others [1,6-8].

Several rheumatic conditions have been associated with the presence of aortitis. Takayasu arteritis (TAK), giant cell arteritis (GCA), long-standing ankylosing spondylitis, Cogan syndrome, and relapsing polychondritis are among the most associated conditions with up to 10% of aortic involvement [2,12-14]. Other connective tissue diseases in which aortitis is less common include rheumatoid arthritis, Behcet's disease, systemic lupus erythematosus, among others [14].

Our patient’s presentation did not have any symptoms or signs that could be easily associated with TAK or GCA. Furthermore, the lack of other vessel involvement, which was supported by the clinical examination, a CT angiography of the chest, abdomen and pelvis, a four-limb arterial doppler testing, and an FDG-PET scan confirmed this as a case of isolated aortitis. Making a distinction between TAK and GCA is difficult when classic clinical features are not present [2,15]. Furthermore, idiopathic aortitis (IA) has been also considered to be part of the disease spectrum encompassing large vessel vasculidites, with IA patients over 50 or 60 years old often classified as GCA [6,12]. However, we believe that using the age to differentiate between TA and GCA would be insufficient to make a final diagnosis in our case.

Other pertinent laboratory results are shown in Table 2 including a negative rheumatoid factor, cyclic citrullinated peptide antibody, negative antinuclear antibodies by immunofluorescence and dsDNA antibody, negative extractable nuclear antigens, negative cytoplasmic neutrophil antibodies, normal levels of complement C3 and C4, and normal levels of immunoglobulin subclass IgG4. In the absence of other clinical manifestations, systemic lupus erythematosus (SLE), rheumatoid arthritis, and antineutrophil cytoplasmic antibodies (ANCA)-associated vasculitides were excluded. Likewise, she lacked other typical manifestations of relapsing polychondritis. Inflammatory eye disease is common in patients with Behcet’s disease and Cogan’s syndrome, but she did not have a history of oral or genital ulcerations or vestibuloauditory dysfunction that would be expected in those conditions.

## Conclusions

In conclusion, our case of isolated abdominal aortitis without aneurysm represents a unique clinical challenge that could be classified as idiopathic. Isolated idiopathic aortitis has been usually described as involving the thoracic aorta, making our case even rarer. Another interesting event in our case is the presence of a urinary tract infection preceding the diagnosis of aortitis. Although we believe that this is a case of non-infectious aortitis, microbial agents can trigger autoimmunity through different mechanisms, contributing to the development of primary systemic vasculidites. Unfortunately, there are no similar cases published and we cannot rule out that the identification of these two concomitant events could have happened by chance based on the silent course of some cases of aortitis. Nonetheless, the proper identification of IA should not be underestimated, since cases of IA have been reported to be more severe with higher proportions of aortic aneurism at diagnosis and worse prognosis in patients under 60 years old. Finally, whether *E. coli* infection can trigger the immune system and result in vasculitis needs further investigation.
